# An exploration of health workers risks of contracting tuberculosis in the workplace: a qualitative study

**DOI:** 10.1186/s12913-020-05877-0

**Published:** 2020-11-12

**Authors:** Shadreck Mwenya, Salley Stapley

**Affiliations:** 1 School of Health and Social Care, Teesside University, Middlesbrough, Tees Valley, TS1 3BX UK; 2 Zambia Ministry of Health (Namwala District Health Office), Lusaka, Zambia

**Keywords:** Health workers, Tuberculosis, Awareness, Health promotion, Qualitative research

## Abstract

**Background:**

To explore the perceptions of health workers on the risks of contracting tuberculosis at Namwala District Hospital. Tuberculosis literature indicates that health workers are at risk of contracting tuberculosis while conducting their daily duties in the workplace. This is mainly attributed to low tuberculosis awareness. It is with this empirical evidence that this study was conducted to further explore health workers risky behavior, attitude and practices that expose them to tuberculosis infection when on duty and eventually generate effective health promotion and public health interventions.

**Methods:**

Semi-structured interviews lasting between 35 to 45 min were conducted to all the participants. A purposive sampling technique was used to recruit ten participants for this study. All the ten interviews were audio recorded in order to enhance consistency during data analysis process. Interview materials were transcribed verbatim, coded and themes generated to form thematic networks. Data analysis was conducted using thematic analysis strategy.

**Results:**

Four themes were identified; 1. *Health workers personal safety*: participants reported wearing uniforms and gloves but they were not putting-on face masks hence, exposing themselves to tuberculosis infection. 2. *Tuberculosis infection prevention practices*: hand washing was described by many participants as a universal method of protecting health staff from the risks of contracting tuberculosis at the hospital however, few health workers frequently washed their hands after attending to tuberculosis patients. 3. *Health workers working environment*: the working environment at the hospital was not conducive for both health workers and patients due to poor ventilation, unhygienic conditions, overcrowding and the lack of an isolation ward. 4. *Health promotion*: awareness on tuberculosis was reported to be low with no refresher training being conducted for health workers at the hospital.

**Conclusion:**

The risks of contracting tuberculosis by health workers at Namwala District Hospital did exist hence, a need of advocating for tuberculosis awareness for health workers through appropriate health promotion interventions. Health policy should focus on continuous health promotion activities on prevention and control of tuberculosis in health facilities and communities.

## Background

Tuberculosis is an airborne infectious disease caused by bacteria called *Mycobacterium tuberculosis* [1]. The burden of tuberculosis still remains a huge public health concern worldwide because it is ranked second in causing millions of deaths following human immunodeficiency virus and it is also responsible for causing ill-health and deformities [2]. Globally, it is estimated that every year approximately 10 million people develop tuberculosis with 1.3 million deaths [3]. Overall, 90% of tuberculosis cases were recorded in the adult population with 9% accounting for individuals infected with human immunodeficiency virus in the year 2017 [4]. Furthermore, 1.7 billion people are estimated to have latent tuberculosis infection with a high possibility of developing active tuberculosis later in their lifetime [5]. Globally, health care professionals are at high risk of contracting tuberculosis than the general population [6]. Additionally, it is estimated that the risk of contracting tuberculosis by health care workers in Zambia is twenty times higher than other individuals [7].

Tuberculosis is recognized as an occupational hazard for health workers [8]. Additionally, a study conducted at a hospital in the Sub-Saharan African region revealed that 12 out of 310 nurses developed active tuberculosis over a two year period after exposure to tuberculosis infection during their professional practice [7, 8]. Although the tuberculosis prevention guidelines were made available in health facilities in Zambia, tuberculosis infection control and prevention practices among health workers remained a huge challenge in reducing tuberculosis mortality and morbidity [8]. Globally, hospitals are normally considered to be the major providers of health care services to tuberculosis patients however, supervision of the national tuberculosis control programme in many African countries is often unsatisfactory [7].

A similar study conducted in Egypt revealed that health care workers were at high risk of contracting tuberculosis from patients due to the use of defective personal protective equipment, poor hand hygiene, working in poorly ventilated places, poor coughing techniques, non-utilization of face masks, dusty environment and lack of adherence to tuberculosis control policy guidelines [9]. The other risk factors that increase the chances of health workers developing active tuberculosis include tobacco smoking, alcohol drinking, HIV infection, chronic kidney problem, extreme aging, diabetes mellitus, cancer, living in a poorly ventilated dwelling and overcrowded environment [9]. Furthermore, poor attitude, lack of adequate tuberculosis knowledge and poor tuberculosis infection prevention practices among health workers contributes to the spread of tuberculosis within the health facility setting [8, 9]. It is with this empirical evidence that this study was conducted to further explore health workers risky behaviors, attitudes and practices that expose them to tuberculosis infection during their professional practice.

## Methods

### Study design, aims and setting

This study was a phenomenological qualitative research design. It aimed to explore health workers’ experiences regarding their attitudes and behaviors while attending to tuberculosis patients. The study also explored health care workers awareness of tuberculosis transmission within their working environment. The research setting was Namwala District Hospital which had a 100 bed capacity with a total of 120 staff. Namwala District Hospital is located in Namwala District of the Southern Province of Zambia and had a population of 116,337 with 19 health clinics and 1 main hospital. Furthermore, Namwala District had 70% livestock farmers, 20% fishermen and 10% civil servants [10]. The District recorded 150 confirmed tuberculosis cases with 24 mortalities in the year 2015 [11]. All suspected tuberculosis patients from health facilities were referred to the main hospital for diagnoses, treatment and hospitalization hence, making the sampling frame more credible for this study [10, 11]. Additionally, the researcher chose Namwala District Hospital because it was easily accessed and therefore, convenient to gather participants for interviews.

### Participants recruitment and characteristics

Doctors and nurses practicing at Namwala District Hospital were the potential participants for this research study. The researcher purposively sampled 1 medical doctor and 9 nurses to make a total of 10 participants. The recruitment of participants was done through a recruitment poster placed at Namwala District Hospital premises. All recruited participants were working at Namwala District Hospital where tuberculosis patients were diagnosed, treated and hospitalized. One participant had a degree in medicine, the other one a degree in nursing and the rest of the participants had diploma qualifications in nursing. Six enrolled participants had worked between 8 and 15 years while the other four participants had worked between 3 and 5 years in tuberculosis departments at the same health facility. All enrolled participants spent approximately 20 h working in tuberculosis departments in a week. Further characteristics of participants included: three females and seven males ranging from 25 to 40 years old.

### Data collection procedure

The researcher collected data between October and November of 2016 from all the ten participants. Furthermore, the researcher conducted ten face-to-face in-depth interviews to all the participants after they signed the consent forms. All the interviews were guided by pilot tested semi-structured interview schedule and were audio recorded in order to ensure consistency during the data analysis process (Table 1). The interviews were conducted at places and times convenient to every participant. The researcher in this study chose interviews over focus group discussion because it was very difficult to gather all participants at the same time. Additionally, the researcher had little experience of conducting focus group discussions. Semi-structured interviews were used because they allowed all participants to be active and more engaged during the interviews hence, provided strength to the overall research outcomes [12]. All interviews lasted between 35 to 45 min long.

### Data analysis

This study used thematic analysis strategy to analyze collected data from all the ten participants. The researcher transcribed verbatim all the ten interview recordings onto a hard copy, conducted line-by-line coding, identified codes, formed themes and created thematic networks in a web-like structure [13]. Thematic analysis included the following six phases: data familiarization, generation of codes, themes identification, reviewing of the themes, naming of the themes and report writing [14]. The data analysis process yielded thirty-nine codes, twelve basic themes, four organizing themes and one global theme (Table 2). These codes were manually selected by the researcher after data familiarization process. The originality of the interview recording was maintained by returning transcribed copies to the participants for counter-checking and verification. The saturation point was reached during the data analysis process because no new ideas emerged from the data [13, 14].

**Table 1:** **Illustration of the interview schedule**

Interview Schedule	
1. Tell me a little about yourself	
a. Name	
b. Age	
c. Occupation	
2. What is your experience of attending to a tuberculosis patient at Namwala District Hospital?	
3. What could be the risks of contracting tuberculosis from patients at Namwala District Hospital?	
4. What has been your experience on isolation of tuberculosis patients at Namwala District Hospital?	
5. How are tuberculosis infectious wastes stored within the hospital setting/ admission wards?	
6. Describe your experience on the disposal of tuberculosis infectious wastes?	
7. What professional practices could you say contributes to the risks of contracting tuberculosis when attending to tuberculosis patients?	
8. What comes to your mind when you know that you are attending to a tuberculosis patient?	
9. How do you describe your personal safety when attending to a tuberculosis patient?	
10. What have you been doing in order to protect yourself when attending to a tuberculosis patient?	
11. What safety measures do you take after attending to a tuberculosis patient?	
12. What are the risk factors of contracting tuberculosis that health workers at Namwala District Hospital are exposed to?	
13. What could be your risk behavior when attending to tuberculosis patients?	
14. Tell me your experience of working in the ward where there are tuberculosis patients?	
15. Briefly explain the infection prevention practices you undertake when attending to a tuberculosis patient?	
16. How do you describe health workers attitudes towards safety precautions especially when attending to a tuberculosis patient?	
17. Some people say it’s dangerous attending to tuberculosis patients, what is your opinion on this statement?	
18. Health workers are considered to be at risk of contracting tuberculosis especially here at the hospital, what is your comment?	
19 What has been your experience regarding ventilation in the wards where tuberculosis patients are admitted?	
20. How spacious are the rooms were tuberculosis patients are admitted?	
21. Overcrowding has been associated with tuberculosis transmission, how is the situation in the rooms were tuberculosis patients are admitted?	
22. Tuberculosis awareness among health workers has been considered to be inadequate, what is your comment?	
23. Finally, what do you think health promoters should do in raising awareness on tuberculosis prevention to health workers?	
**Thank you for your participation!!**

**Table 2:** Illustration of the identified basic themes, organizing themes and global theme

Basic Themes	Organizing Themes	Global Theme
Community perception	Tuberculosis infection prevention practices	Tuberculosis
Isolation ward		
General ward		
Poor hand washing practices	Health workers personal safety	
Inadequate protective wear		
Poor hygiene		
Overcrowding	Health workers working environment	
Dirty environment		
Poor ventilation		
Health workers	Health promotion	
Bedsiders (Relatives)		
Community		

### Ethical approval and consent

This study obtained ethical clearance from the Biomedical Research Ethics Committee of the University of Zambia and the Research Ethics Committee of Leeds Beckett University. All participants’ names remained anonymous and collected information was kept confidential, safe and only used for academic purposes. The originality of the collected data was maintained including usage of direct quotations. In addition, all participants understood the purpose of this study through reading the information sheet and thereafter, were requested to sign the consent forms before being interviewed if they agreed to participate. Furthermore, all participants were at liberty to withdraw from the research study two weeks after data collection if they desired.

## Results

The four organizing themes that were identified after the data analysis process included the following:

1. Health workers personal safety
2. Tuberculosis infection prevention practices
3. Health workers working environment
4. Health promotion

### Organizing theme 1: health workers personal safety

When asked about professional practices that subjected health workers to the risk of contracting tuberculosis while carrying-out their duties, four participants acknowledged that the majority of nurses who were in frequent contact with tuberculosis patients at the hospital were not wearing face masks because of existing tradition and culture which stigmatize health care workers putting-on face masks. Community members and patients felt discriminated against when a health worker wore a face mask while attending to tuberculosis client. They admitted that the practice compromised their personal safety and exposed them to tuberculosis:

*“...when it comes to attending to these patients, there are those disposable face masks but because of our tradition…most of them do not wear those masks. Very few put on those masks as they are examining those patients, that’s one thing I have seen…”* [Senior Nursing Officer]

The quotation above significantly showed the relationship between tuberculosis control measures and cultural norms. According to the nurses, wearing face masks when attending to tuberculosis patients was against the local culture and tradition. Additionally, patients and community members felt discriminated against when doctors and nurses wore face masks.

Despite six participants confirming the use of disposable gloves during their professional practice; other nurses highlighted that they did not wear disposable gloves all the time that they attended to tuberculosis patients at the hospital. They felt that the tendency of not always wearing disposable gloves when handling and examining tuberculosis patients made them vulnerable to contracting tuberculosis:

*“...health workers do not always put-on disposable gloves when they are attending to tuberculosis patients…”* [Registered Nurse]

According to the above quotation, health workers who were not frequently wearing disposable gloves were at high risk of contracting tuberculosis while performing their duties. Additionally, other nurses had some awareness on tuberculosis transmission and described it to be very infectious and contagious. Furthermore, they also described the risk of contracting tuberculosis at the hospital to be very high:

*“The risks are there…we know that people who have tuberculosis are infectious, very contagious, so the risk is very high”* [Registered Specialist Nurse]

The above quotation reaffirms the significance of health workers wearing complete protective equipment when attending to tuberculosis patients and other highly infectious conditions in order to prevent transmission within the hospital setting. Participants were scared of people with tuberculosis; this resulted in social exclusion and discrimination. Furthermore, one participant echoed that tuberculosis was highly infectious and that it was not only found at the hospital but also in public places and communities:

*“...because even in taxi, mini-buses you can get it, so hmmm, I think we need very serious measures on how to protect health workers and the general public”* [Registered Specialist Nurse]

It is with such evidence that participants felt the need of providing adequate ventilation system not only in public service vehicles but also in dwelling houses and congested places in order to reduce the risk of tuberculosis transmission from an infected person to a healthy individual. Furthermore, two participants responded in a similar way when asked to describe the personal safety of health workers at the hospital:

*“I describe personal safety not to be adequate at our hospital…when you put-on uniform, you can move from one ward to another and yet putting-on the same uniform…”* [Registered Nurse]

The above quotation highlighted on the importance of health workers adherence to personal safety procedures especially when attending to tuberculosis patients. According to the participants, adherence to personal safety procedures within the hospital setting reduced the risks of tuberculosis transmission from patients to health workers and communities.

### Organizing theme 2: tuberculosis infection prevention practices

When asked about the professional practices that contributed to the risks of contracting tuberculosis from patients at the hospital, four participants acknowledged that nurses did not frequently wash their hands after attending to tuberculosis patients:

*“...not taking frequent hand washing and sometimes you find that nurses are consuming food just in the ward where they are attending to tuberculosis patients”* [Registered Nurse]

Participants recognized hand washing as a universal personal hygiene practice and best infection prevention strategy that could reduce transmission of tuberculosis within the hospital setting and communities. Furthermore, participants confirmed that the hospital had no isolation ward for the admission of confirmed tuberculosis patients; instead tuberculosis patients were admitted together with other patients with different medical conditions in the same ward:

*“The risks of tuberculosis are there because we do not have an isolation ward, we just combine them in the main ward, so I would just say that the risk is 100%, it is just by the grace of God that we are not infected”* [Enrolled Nurse]

The above quotation revealed that there were compromised tuberculosis prevention practices at the hospital. Furthermore, inadequate staffing was such a big issue that one participant linked it to compromised tuberculosis prevention practices by health workers at the hospital:

*“We do not have nurses specifically for tuberculosis ward, so the practice has been that if the emergency happens, you do not even take-off what you have been using in the tuberculosis ward or rather the uniform or whatever gear you have been putting-on and go straight to the other wards. That is actually what has compromised infection prevention, its inadequate staffing…”* [Registered Nurse]

Additionally, two participants responded in a similar way when asked to describe the risk behaviors of health workers after attending to tuberculosis patients at the hospital:

*“...you go with your clothes back home, it’s not like in western countries were you will have a locker of uniforms and coats but here you go with them home, if you have carried the bacterium home, even the children will get it, so those I think can be risk behaviors”* [Registered Specialist Nurse]

The above quotation showed how tuberculosis could be transmitted from the hospital setting to communities. However, participants suggested that key tuberculosis preventive health messages should not only be disseminated to health workers but also to the general population. In addition, when asked what came into their mind when attending to tuberculosis patients at the hospital, one participant linked tuberculosis to HIV/AIDS infection:

*“What comes to my mind hmmm, [Laughs], obviously it’s hmmm HIV comes there, looking at the way they are related…”* [Enrolled Nurse]

The above quotation clearly showed how health workers and community members stigmatize patients with tuberculosis. According to the majority of participant’s views, people with tuberculosis were perceived to be very infectious and socially excluded. Furthermore, participants linked health workers fear of tuberculosis patients to the health inequalities observed in health facilities and communities. In addition, one participant confirmed that health workers at the hospital treated tuberculosis patients like any other patient without considering infection prevention interventions hence, exposing themselves to the risks of contracting tuberculosis while on duty:

*“A lot of people come with cough but you just take it to be a normal cough…”* [Registered Specialist Nurse]

Furthermore, another participant confirmed that storage of tuberculosis infectious wastes within the wards at the hospital was not according to the recommended standards due to limited waste bins hence, resulted in non-segregation of infectious wastes:

*“We just throw them in the bin and there is only one bin which we use to keep wastes from all patients. Hmmm, there are no bins specifically meant for tuberculosis wastes”* [Registered Nurse]

The quotation above significantly indicated that there was compromised health care waste management at the hospital thereby, contributing to the risks of health workers contracting infectious diseases including tuberculosis during their professional practice. Therefore, participants felt the need of providing adequate waste bins and segregation of infectious wastes in the wards in order to prevent the risks of contracting tuberculosis at the hospital.

### Organizing theme 3: health workers working environment

According to three participant’s views, the working environment at the hospital was not conducive to the health workers and that it favoured transmission of tuberculosis:

*“Hmmm, I would say yes because when you look at the conditions we are working-in, it favours transmission of tuberculosis”* [Registered Nurse]

Additionally, another participant acknowledged the importance of working in a clean and conducive environment and that it helps reduce the transmission of infectious diseases. She also mentioned that it would be quiet difficult for *Mycobacterium tuberculosis* to flourish in a clean hospital setting:

*“...then also even the environment where you are treating those patients, it has to be very clean, it has to be dump dusted, it has to be cleaned, so that the chances of the bacterium to be in the environment is reduced hence, even reducing the chances of transmission”* [Registered Specialist Nurse]

The majority of participants acknowledged that the working environment at the hospital facilitated the transmission of tuberculosis from patients to health care workers and eventually to other community members. Furthermore, when asked to describe their experiences regarding ventilation in the wards where tuberculosis patients were admitted, five participants gave a similar response:

*“...there is no much space for adequate ventilation, the rooms are small and not well ventilated in short”* [Nurse]

Participants confirmed that the rooms where tuberculosis patients were admitted were too small and poorly ventilated. In addition, five participants gave a similar response when asked to explain whether tuberculosis patients were overcrowded in the admission wards:

*“...as I speak now when you go to female ward you will find that the ward is fully packed and what if there is one who is being investigated for tuberculosis, then they are crowded, that’s already the risk to the patients even to the health workers who are there”* [Registered Specialist Nurse]

Furthermore, another participant acknowledged that there were many people visiting the wards where tuberculosis patients were admitted at the hospital:

*“My experience has been that hmmm, in our setting there has been no restriction whatsoever of people visiting, so traffic to these wards has not been controlled, so it is very easy for people to catch infections”* [Registered Nurse]

Many participants felt that congestion and overcrowding in the wards where tuberculosis patients were admitted was caused by non-restriction of people visiting the wards.

### Organizing theme 4: health promotion

Five participants responded in a similar way when asked whether tuberculosis awareness among health workers was adequate:

*“Hmmm, I can say it is somehow inadequate because hmmm, those messages for awareness of tuberculosis are kept in the book, they are not really disseminated to everyone…”* [Medical Doctor]

Additionally, one participant said that due to inadequate tuberculosis awareness, two nurses had already contracted tuberculosis from patients and were admitted and receiving treatment at the same hospital:

*“...we even have members of staff on the ward who have contracted tuberculosis and are on treatment, two nurses actually. That shows that there is a problem”* [SeniorNursing Officer]

Furthermore, one participant revealed that lack of health education sessions to relatives of patients admitted at the hospital was another professional practice that contributed to the risks of tuberculosis transmission from patients to health workers:

*“Sometimes hmmm, no proper health education is given to bedsiders or relatives to the patients…”* [Registered Nurse]

In addition, another participant highlighted that there were inadequate tuberculosis prevention materials distributed to health workers, relatives to the patients and the general population:

*“Hmmm, they should provide them with enough health education materials to use when they are teaching tuberculosis patients on how to cough and also on the principles of tuberculosis infection prevention”* [Nurse]

Again, one participant felt the need of having children vaccinated against tuberculosis:

*“...so I would educate these patients on the need of having their children vaccinated against tuberculosis…”* [Enrolled Nurse]

Furthermore, two participants revealed that there were no refreshers training on tuberculosis held at the hospital especially for the newly recruited health workers:

*“...there have been no tuberculosis refresher training for health workers, hmmm there have been no new skills on prevention of tuberculosis especially to the health workers who have just started working…”* [Registered Nurse]

Additionally, another participant felt the need of extending health promotion on the coughing techniques to patients and advised them to properly cover their mouth when coughing as a way of preventing tuberculosis transmission to health workers and the general public:

*“Hmmm, the first thing, I think is to educate patients that hmmm, on how they should handle their cough, I mean covering their mouth whenever they are coughing…because these tuberculosis patients just cough and spit anyhow”* [Enrolled Nurse]

Furthermore, one participant echoed the significance of having effective health promotion strategies on tuberculosis prevention and noted that he would like to see a health promoter conduct tuberculosis training workshop to health workers and also within communities:

*“Hmmm, mostly like doing tuberculosis workshop, I would like to see someone come to teach more about tuberculosis and educate health workers so that they become aware hmmm, workshops, sensitization even in the communities so that communities can be aware that tuberculosis is there, it’s real, it’s curable and preventable”* [Tuberculosis Coordinator]

In addition, one participant suggested that it would be more effective for health promoters to raise tuberculosis awareness to health workers and the general public during world tuberculosis day commemoration because the disease affected everyone indirectly or directly:

*“I think during world tuberculosis day commemoration, they should involve much of health workers but also ordinary individuals because you find that, like the current situation, the hospital is not involved much in the tuberculosis awareness programmes…so it makes them to be vulnerable to tuberculosis…”* [Senior Nursing Officer]

Furthermore, another participant suggested an effective method of disseminating key tuberculosis awareness messages to both health workers and patients:

*“...so they need to be reminded, you can even put pictures because with pictures it is very easy for someone to pick the message and not to forget, then it will remain in their mind and people will continue spreading the message in that way”* [Medical Doctor]

According to the above quotation, participants felt that disseminating tuberculosis awareness messages to the targeted audience through pictures was more effective because the picture messages always remained in their mind.

## Discussion

The findings of the current study show significant evidence that health professionals working at Namwala District Hospital are at high risk of contracting tuberculosis while on duty. This is supported by one participant who revealed that two nurses at Namwala District Hospital had already contracted tuberculosis during their professional practice and were admitted and treated at the same hospital. Additionally, this study found lack of an isolation ward, non-usage of face masks and gloves, unhygienic working conditions, overcrowding, poor ventilation and inadequate tuberculosis prevention practices as major contributing factors for tuberculosis transmission from patients to health workers at the hospital. These findings are consistent with a similar study by De Perio & Niemeier (2014) who observed that there was an increased risk of tuberculosis transmission when health workers practiced inadequate tuberculosis prevention strategies [6]. However, another study by Tiemersma et al. (2016) found that availability of infection prevention focal point person in health facilities encouraged health workers adherence to infection control principles and thereby reduced the risk of contracting tuberculosis and other infectious diseases when on duty [15].

Additionally, the current study found that nurses working at Namwala District Hospital had poor attitudes towards infectious disease prevention because they were not frequently wearing disposable hand gloves and face masks during their professional practice. Furthermore, this study noted that health workers at Namwala District Hospital were not frequently washing their hands after attending to tuberculosis patients hence, increased the chances of contracting tuberculosis and other infectious diseases within the hospital setting [16]. These findings are similar to another study carried-out in Jordan which found that nurses had inadequate knowledge of personal safety procedures hence, needed to be trained in tuberculosis infection prevention and personal safety practices [17].

Furthermore, a study by Dokubo et al. (2016) recommended that hospital administrators should ensure that managerial, supervision and environmental controls are strengthened in order to promote effective personal safety practices by every staff [18]. Again, similar research suggested that the Ministry of Health should develop and implement policies aimed at prevention and control of occupational tuberculosis thereby, improving the lives of health workers and the general population however, very few health facilities have successfully implemented tuberculosis infection control procedures [7].

Interestingly, the current study found that health workers at Namwala District Hospital were taking the cough of suspected tuberculosis patients like a normal cough without taking the necessary safety procedures despite them describing tuberculosis to be a very dangerous and contagious disease. Furthermore, this study revealed that the admission rooms where confirmed tuberculosis patients were admitted at the hospital were very small, overcrowded, poorly ventilated and with weak restriction control measures for visitors. These findings are consistent with another study by Cochrane (2014) which found that there was poor hand washing practices, no cough hygiene, poor ventilation, overcrowding and non-existence of tuberculosis infection control measures in the majority of health care facilities where tuberculosis patients were diagnosed, treated and admitted [19].

Additionally, another study conducted in Brazil found that inadequate staffing at hospitals contributed to poor tuberculosis infection control procedures by available health staff hence; they recommended that adequate staff be employed through healthy public policy [20]. It is from such overwhelming evidence that health promoters should tailor their interventions towards addressing the wider environmental, economic and social determinants of health as underpinned by the concept of upstream thinking [21].

According to a study conducted in Tajikistan, the risk factors associated with tuberculosis transmission include poverty, poor housing, overcrowding, social exclusion, inadequate knowledge and low social economic status and that the prevalence was high among health care workers [22]. In contrast, a similar study done in Italy among medical students found low transmission of latent tuberculosis however, the authors noted that there was high occupational risk of tuberculosis infection among participants even in countries with low prevalence of tuberculosis [23]. Additionally, the current study found that tuberculosis patients at the hospital were coughing and spitting anyhow, non-segregation of tuberculosis wastes and the environment favoured transmission of tuberculosis to health workers and the general population. Furthermore, this study observed that there was inadequate tuberculosis awareness activities for health workers and patients at the hospital; this was evidenced when participants revealed that there were no tuberculosis refresher training to newly recruited staff and no health promotion materials on the coughing techniques were distributed to patients and their relatives.

It is from such findings that health promoters and public health experts are encouraged to develop and implement health policies aimed at preventing and controlling tuberculosis not only among health workers in health facilities but also focus on the entire population within communities in order to enhance health and well-being [24]. In addition, psychological empowerment is known to increase individuals’ self-esteem, self-efficacy and confidence in order for people to take control over their own health however, it is limited because it does not tackle the wider social determinants of health [25]. Furthermore, the World Health Organization recommended that health promoters should endeavor to create supportive environments in order to enhance the health of individuals and communities [26].

According to Mirtskhulava et al. (2015) the health belief model is another evidence-based behavior change model that could be utilized by public health and health promotion practitioners to influence behavior change by focusing on perceived severity, perceived susceptibility, perceived benefits, perceived barriers and cues to action [27]. In addition, the World Health Organization approved the end tuberculosis strategy however, the global decline of tuberculosis incidence remained low in 2016 at 1.5% but this should be accelerated to 5% by the year 2020 in order to effectively fight and end tuberculosis [28]. Furthermore, a study conducted in Peru found that tuberculosis incidence drastically reduced in 1990 from 532/100,000 to 95/100,000 by the year 2012 due to effective implementation of health promotion strategies; although lack of community participation and engagement hindered the tuberculosis control programme [29]. Therefore, public health and health promotion practitioners are encouraged not only to raise tuberculosis awareness but also to implement healthy public policy that tackles socioeconomic, cultural and environmental aspects that enhance health and well-being [30].

### Limitations of the study

This study found limited research work conducted in similar settings as reference material. Observing participants is more likely to produce accurate results in qualitative studies than investigating practice using a semi-structured interview schedule, but this study did not collect data through observations.

## Recommendations

The following recommendations are provided based on the findings of this research:

1. Health workers should improve tuberculosis prevention practices by frequently wearing disposable hand gloves, face masks and ensure that they regularly wash their hands with soap after attending to tuberculosis patients.
2. Health workers should ensure that they take the necessary safety precautions when attending to any suspected tuberculosis case.
3. Provide an isolation ward, spacious admission rooms, avoid overcrowding and ensure that the admission rooms for tuberculosis patients are kept clean and properly ventilation in order to reduce the risks of tuberculosis transmission within health facilities.
4. Disseminate key tuberculosis awareness messages to health workers and patients in health facilities and communities.
5. Provide adequate tuberculosis waste bins and ensure that there is segregation of infectious and non-infectious wastes in health facilities.
6. Conduct tuberculosis refresher training to newly recruited health staff in health facilities.
7. Quantitative studies should be carried-out in health facilities in order to quantify health workers who could have contracted tuberculosis during their professional practice.

### Implications for practice

The findings of this research has the potential to influence health managers, hospital administrators and policy makers to strengthen health promotion and public health interventions that are tailored on tuberculosis prevention and awareness for the general public and health workers and ultimately contribute to the end of the tuberculosis global strategy.

## Conclusion

The findings of the current study demonstrated significant evidence that health workers practicing at Namwala District Hospital were at high risk of contracting tuberculosis from patients during their professional practice. The risk factors that facilitated tuberculosis transmission from patients to health workers at the hospital included; overcrowding, poor ventilation, absence of an isolation ward, small admission rooms, poor coughing techniques, inadequate personal protective equipment, inadequate tuberculosis infection prevention practices and unhygienic conditions. However, public health and health promotion practitioners could utilize health promotion interventions that are underpinned by fear appeals such as the health belief model to influence the behavior change of health workers and ordinary individuals and thereby contribute effectively to the end of the tuberculosis global strategy.

## Data Availability

The anonymized datasets used and/or analyzed during the current study are available from the corresponding author on reasonable request.

## Abbreviations

HIV: Human immunodeficiency virus
AIDS: Acquired immune deficiency syndrome

## References

[1] Vorster MJ, Allwood BW, Diacon AH, Koegelenberg CFN (2015). Tuberculosis pleural effusions: advances and controversies. Journal of Thoracic Disease.

[2] Tola HH, Tol A, Shojaeizadeh D, Garmarouchi G (2014). Tuberculosis treatment non-adherence and lost to follow-up among TB patients with or without HIV in developing countries: a systematic review. Iran Journal of Public Health.

[3] World Health Organization (2018). Global Tuberculosis Report.

[4] MacNeil A, Glaziou P, Sismanidis C, Maloney S, Floyd K (2019). Global epidemiology of tuberculosis and progress toward achieving global targets-2017. Morb Mortal Wkly Rep.

[5] Morano JP, Zelenev A, Walton MR, Bruce D, Altice FL (2014). Latent tuberculosis infection screening in foreign-born populations: a successful mobile clinic outreach model. Am J Public Health.

[6] De Perio MA, Niemeier RT (2014). Evaluation of exposure to tuberculosis among employees at a medical Centre. J Occup Environ Hyg.

[7] Da Silva EH, Padoveze MC, Tanaka AT, Higa RCM, Americo LGC (2015). Evaluation of occupational tuberculosis prevention in a Brazilian hospital. Rev Rene.

[8] Abdullah S, Ismail I, Nor SSM, Wahab FA (2014). Reliability of knowledge, attitude and practice (KAP) questionnaire on tuberculosis among health workers (HCWS). Int Med J.

[9] Salleh SFM, Rahman NA, Rahman NI, Haque M (2018). Knowledge, attitude and practice towards tuberculosis among community of Kulim municipal council, Kedah, Malaysia. Int Med J.

[10] Central Statistics Office (2010). Zambia Census of Population and Housing, Population Summary Report, Central Statistics Office, Lusaka.

[11] Malagon-Maldonado G (2014). Qualitative research in health design. Health Environ Res Design J.

[12] McCusker K, Gunaydin S (2015). Research using qualitative, quantitative or mixed methods and choices based on the research. Perfusion.

[13] Palmer AM, Correa JB, Heckman BW, Brandon TH, Simmons VN (2016). Health, stigma and the burden of smoking in college: a thematic analysis. Am J Health Behav.

[14] Vargas RM, Mahtani-Chugani V, Pallero MS, Jimenez BR, Dominguez RC, Alonso VR (2016). The transformation process for palliative care professionals: the metamorphosis, a qualitative research study. Palliat Med.

[15] Tiemersma EW, Huong NT, Yen PH, Tinh BT, Thuy TTB, Hung NV, Mai NT, Verver S, Gebhard A, Nhung NV (2016). Infection control and tuberculosis among health care workers in Viet Nam. BMC Infect Dis.

[16] Murry MT, Jackson O, Cohen B, Hutcheon G, Saiman L, Larson E, Neu N (2016). Impact of infection prevention and control initiatives on acute respiratory infections in a pediatric long-term care facility. Infect Control Hospital Epidemiol.

[17] Saleh AM, Darawad MW, Al-Hussami M (2015). The perception of hospital safety culture and selected outcomes among nurses: an exploratory study. Nursing Health Sci.

[18] Dokubo EK, Odume B, Lipke V, Muianga C, Onu E, Olutola A, Ukachukwu L, Igweike P, Chukwura N, Ubochioma E, Aniaku E, Ezeudu C, Agboeze J, Iroh G, Orji E, Godwin O, Raji HB, Aboje SA, Osakwe C, Debem H, Bello M, Onotu D, Maloney S (2016). Building and strengthening infection control strategies to prevent tuberculosis-Nigeria, 2015. Morb Mortal Wkly Rep.

[19] Cochrane C. A voluntary experience in Bairo Pite hospital, Timor Leste. Queensland Nurse. 2014;33(2):39. 24791330

[20] Tobias GC, Bezerra ALQ, Paranagua TTD, Silva AEBD (2016). Safety culture in a teaching hospital: strength and weaknesses perceived in nurses. J Nursing UFPE.

[21] Davis SL, Chapa DW (2015). Social determinants of health: knowledge to effective action for change. J Nurse Pract.

[22] Gilpin C, De Colombani P, Hasanova S, Sirodjiddinova U. Exploring TB-related knowledge, attitude, behavior and practice among migrant workers in Tajikistan. Tuberculosis Res Treatment. 2011;1:1–10. 10.1155/2011/548617 PMC3335497 22567266

[23] Durando P, Alicino C, Orsi A, Barberis I, Paganino C, Dini G, Mazzarello G, Del Bono V, Viscoli C, Copello F, Sossai D, Orengo G, Sticchi L, Ansaldi F, Icardi G. Latent tuberculosis infection among a large cohort of medical students at a teaching hospital in Italy. Biomed Res Int. 2015;1:1–6. 10.1155/2015/746895 PMC4331323 25705685

[24] Tang KC, Stahl T, Bettcher D, De Leeuw E (2014). The eight global conference on health promotion: health in all policies: from rhetoric to action. Health Promot Int.

[25] Taghipour A, Borghei NS, Roudsari RL, Keramat A, Nooghabi HJ (2016). Psychological empowerment model in Iranian pregnant women. Int J Community Based Nursing Midwifery.

[26] World Health Organization (2009). Milestones in Health Promotion: Statements from Global Conferences.

[27] Mirtskhulava V, Whitaker JA, Kipiani M, Harris DA, Tabagari N, Owen-Smith AA, Kempker RR, Blumberg HM (2015). Determinants of tuberculosis infection control-related behaviors among healthcare workers in the county of Georgia. Infect Control Hosp Epidemiol.

[28] Hu Y, Zhao Q, Wu L, Wang W, Yuan Z, Xu B (2013). Prevalence of latent tuberculosis infection and its risk factors in schoolchildren and adolescents in Shanghai, China. Eur J Public Health.

[29] Ivany E, Boulton J (2014). Challenges in tuberculosis management in Peru and England. Br J Nurs.

[30] Bunton R, Nettleton S, Burrows R (2005). The sociology of health promotion; critical analysis of consumption, lifestyle and risk.

